# Comparison Between RIRS and Mini-PCNL in the Treatment of Kidney Stones Exceeding 15 mm: Outcome Evaluation and Cost Analysis

**DOI:** 10.3390/jcm15010177

**Published:** 2025-12-26

**Authors:** Paolo Pietro Suraci, Andrea Fuschi, Manfredi Bruno Sequi, Fabio Maria Valenzi, Alice Antonioni, Onofrio Antonio Rera, Yazan Al Salhi, Damiano Graziani, Giorgio Martino, Giuseppe Candita, Filippo Gianfrancesco, Paolo Benanti, Cosimo De Nunzio, Giorgio Bozzini, Michele Di Dio, Pierluigi Russo, Matteo Pacini, Carlo Introini, Antonio Carbone, Antonio Luigi Pastore

**Affiliations:** 1 Urology Unit, Department of Medico-Surgical Sciences and Biotechnologies, Faculty of Pharmacy and Medicine, Sapienza University of Rome, Via Franco Faggiana 1668, 04100 Latina, Italy; spaolopietro@gmail.com (P.P.S.); andrea.fuschi@uniroma1.it (A.F.); mb.sequi@gmail.com (M.B.S.); fabiomaria.valenzi@uniroma1.it (F.M.V.); alice.antonioni@gmail.com (A.A.); onofrioantonio.rera@uniroma1.it (O.A.R.); yazan5585@gmail.com (Y.A.S.); damiano.graziani@uniroma1.it (D.G.); giorgio.martino@uniroma1.it (G.M.); giuseppe.candita@uniroma1.it (G.C.); filippo.gianfrancesco@uniroma1.it (F.G.); paolo.benanti@uniroma1.it (P.B.); antonio.carbone@uniroma1.it (A.C.); 2 Department of Urology, Sant’Andrea Hospital, Sapienza University of Rome, Via di Grottarossa 1035/1039, 00189 Rome, Italy; cosimo.denunzio@uniroma1.it; 3 Department of Urology, ASST Lariana-Sant’Anna Hospital, Via Ravona 20, San Fermo della Battaglia, 22042 Como, Italy; gioboz@yahoo.it; 4 Division of Urology, Department of Surgery, Annunziata Hospital, 87100 Cosenza, Italy; m.didio@aocs.it; 5 Department of Urology, IRCCS A. Gemelli University Polyclinic Foundation, 00168 Rome, Italy; pierluigi.russo@guest.policlinicogemelli.it; 6 Department of Life Science, Health, and Health Professions, IRCCS A. Gemelli University Polyclinic Foundation, 00168 Rome, Italy; 7 Urology Unit, Department of Translational Research and New Technologies in Medicine and Surgery, University of Pisa, 56126 Pisa, Italy; matteopacini93@gmail.com; 8 Urology Unit, Department of Abdominal Surgery, E.O. Ospedali Galliera, 16128 Genoa, Italy; carlo.introini@galliera.it

**Keywords:** kidney stones, urolithiasis, retrograde intrarenal surgery, mini-percutaneous nephrolithotomy, RIRS, mini-PCNL, cost analysis, stone-free rate, micro-costing, length of stay, operative time, endourology, health economics, real-world data

## Abstract

**Background/Objectives:** The optimal surgical approach for kidney stones (KS) measuring 15–20 mm remains debated. RIRS and mini-PCNL are both effective options, but they differ in invasiveness, resource use, and cost. This study aimed to compare perioperative outcomes and hospital costs of RIRS and mini-PCNL using a micro-costing approach. **Methods:** This retrospective study included patients with KS > 15 mm in diameter who were treated between January 2021 and December 2023 at the Department of Urology, Sapienza University of Rome-Polo Pontino. Clinical parameters, operative time (OT), length of stay (LoS), complications, and stone-free rate (SFR) were compared. Costs were estimated using a micro-costing method, including disposable materials, operating room (OR) time (3.9 EUR/min), imaging, and hospitalization (334 EUR/day). The total cost per treated and per SF patient was calculated for both techniques. **Results:** A total of 119 patients were analyzed: 62 underwent RIRS, and 57 underwent mini-PCNL. Mean OT was shorter for RIRS (87 vs. 113 min; *p* < 0.001), and LoS was longer for mini-PCNL (2.24 vs. 1.22 days; *p* = 0.008). Final SFR was higher for mini-PCNL (94.7% vs. 88.7%; *p* = 0.043). Complication rates were comparable, with most events classified as Clavien–Dindo I–II. Disposable materials represented the main cost driver (EUR 1097 for RIRS vs. EUR 806 for mini-PCNL). The total cost per treated patient was EUR 3689 for RIRS and EUR 3154 for mini-PCNL (*p* = 0.009). The cost per SF patient was EUR 4159 for RIRS and EUR 3331 for mini-PCNL (*p* = 0.007). **Conclusions:** Both RIRS and mini-PCNL are safe and effective for the management of KS ≥ 15 mm. Mini-PCNL achieves higher SFR and greater cost-efficiency than RIRS. These findings support the use of mini-PCNL as the preferred option in centers with adequate expertise and resources.

## 1. Introduction

Urolithiasis, in general, including kidney stones (KS), is among the most common and ancient urinary tract pathologies, representing a significant public health concern [1]. The incidence of kidney stones depends on geographical, climatic, ethnic, dietary, and genetic factors. In Italy, new cases are estimated to be around 100,000 per year [2,3]. In the general population, KS affects approximately 10% of males and 5% of females, with the majority occurring between ages 30 and 50, leading to significant complications and discomfort. In countries with a high Human Development Index (HDI), such as Sweden, Canada, or the United States, the prevalence of kidney stones is exceptionally high and continues to increase [1,4,5,6]. The main techniques used to treat urinary stones are extracorporeal shock wave lithotripsy (ESWL), percutaneous nephrolithotomy (PCNL), and retrograde endoscopic interventions, including ureteroscopy (URS) and retrograde intrarenal surgery (RIRS). Indications for laparoscopic and robot-assisted surgery remain for the treatment of large “staghorn” stones associated with concurrent need for urinary tract reconstruction [i.e, pyelo-ureteral junction (PUJ) stenosis] [7]. According to the European Association of Urology (EAU) guidelines, PCNL is preferable to RIRS or ESWL for stones larger than 20 mm. Conversely, RIRS or ESWL may be preferred for stones smaller than 10 mm [8]. However, for those with diameters between 10 and 20 mm, there is no definitive preference between PCNL and RIRS. According to the American Urological Association (AUA) guidelines, urologists should propose PCNL as the first-line therapy for overall stone diameters > 20 mm (Dmax); otherwise, RIRS might be considered [9]. Treatment options varied widely among surgeons, facilities, and even across countries and continents. It is also well known that the diagnosis-related group (DRG) system assigns RIRS and PCNL to different reimbursement levels within the National Health Service, favoring the latter [10]. In an era where optimizing expenditures and excellence in patient care are crucial, it is essential to consider the actual economic value of patient care. This study aims to compare RIRS and mini-PCNL with respect to perioperative outcomes and associated costs in treating KS ≥ 15 mm.

## 2. Materials and Methods

### 2.1. Study Design, Population, and Data Collection

The study was conducted retrospectively by examining the database and medical records of patients who underwent RIRS and mini-PCNL for kidney stones between January 2021 and December 2023 at the Department of Urology of Sapienza University of Rome-Polo Pontino, ICOT, in Latina. Up to April 2022, PCNL procedures were not performed in our department; therefore, patients treated during this period underwent RIRS exclusively. After the introduction of the mini-PCNL program, both procedures were available, and the choice of technique was primarily determined by the availability of trained personnel and equipment rather than specific stone or patient characteristics. The study was conducted in accordance with the Declaration of Helsinki and was approved by the Institutional Review Board (UROUNIVLT_OCT20/5821 approved on 12 October 2020). Inclusion criteria were as follows: age ≥ 18 years; and patients with a single KS or multiple adjacent calculi with a Dmax ≥ 15 mm. Patients undergoing concurrent ureteral lithotripsy, those with multiple non-contiguous KS, those with bilateral KS, those with kidney and/or ureter abnormalities, and those with incomplete data were excluded from the study. All patients underwent preoperative abdominal computed tomography (CT) and urine examination with culture. Patients received antibiotic therapy for preoperative infection based on antibiogram results and sensitivity testing. Expert surgeons (with >than 150 cases in both RIRS and mini-PCNL) performed all procedures (P.P.S., A.F., and Y.A.S.). The population was divided into two groups based on the treatment received: Group A (RIRS) and Group B (mini-PCNL). For each patient, the following data were collected: age, gender, body mass index (BMI), preoperative and 24 h postoperative hemoglobin (Hb), preoperative and 24 h postoperative serum creatinine, preoperative and postoperative leukocyte count (white blood cells, WBC), preoperative pharmacological therapy, comorbidities, number of stones, stone Dmax, location and density of stones assessed by CT in Hounsfield units (HU), renal anomalies, operative time (OT), number of procedures per patient, length of hospital stay (LoS), and complications according to Clavien–Dindo classification. Patients were re-evaluated 30 days after the procedure using non-contrast CT of the abdomen and pelvis to assess the stone-free rate (SFR). Absolute SFR was defined as the complete absence of visible residual fragments. SFR included clinically insignificant residual fragments (CIRF), which were the presence of residual fragments ≤ 4 mm. This definition was used as the primary outcome measure.

### 2.2. Surgical Technique and Instrumentation Used in RIRS

The procedure was performed as a one-day surgery based on the patient’s clinical condition. Under general anesthesia, the patient is placed in the lithotomy position. The bladder is accessed directly with a semi-rigid 7.5 Fr ureteroscope. An exploratory ureteroscopy is performed after inserting a Nitinol guidewire (Sensor-Boston Scientific, Marlborough, MA, USA) coated in polytetrafluoroethylene (PTFE) and designed for tactile feedback with a hydrophilic tip in the ureter. A10/12 Fr Rocamed dual-lumen ureteral is positioned under fluoroscopic guidance on the guidewire. The disposable flexible ureteroscope (PUSEN^®^ 7.5 Fr, Johns Creek, GA, USA) was used with Holmium Laser (Lumenis Pulse™ 100H, Yokneam, Israel) to perform lithotripsy. The stone is thus fragmented/pulverized after the laser is set according to the characteristics of the stone formation. A 365 µm laser fiber was routinely employed, whereas a 200 µm fiber was preferred for lower calyceal stones to enhance deflection and maneuverability. At the end of the procedure, a double-J stent is placed, and a 16/18 Fr Foley catheter is placed, which will be removed on postoperative day (POD) 1.

### 2.3. Surgical Technique and Instrumentation Used in the Mini-PCNL

**Figure 1:**
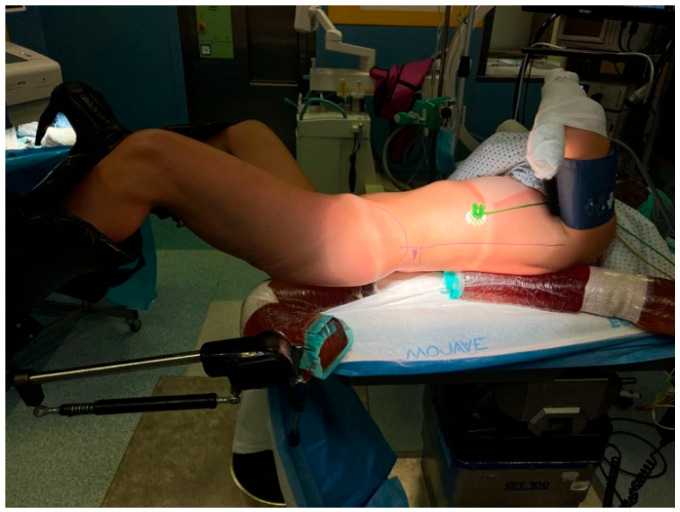
Patient positioning for mini-PCNL in the modified Valdivia–Galdakao supine position.

The procedure was performed during routine admission, with discharge on POD2. Under general anesthesia, the patient is then positioned supine in the modified Valdivia–Galdakao position (Figure 1).

**Figure 2:**
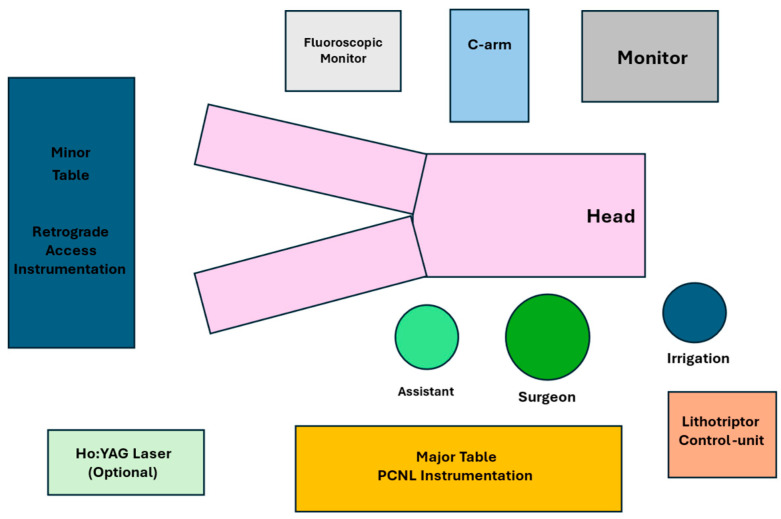
Schematic representation of the operating room setup for mini-PCNL.

The typical OR setup is illustrated in Figure 2. Two instrument tables are prepared: a “lower” table and an “upper” table. The lower table contains all equipment required for retrograde access, including a single sterile drape, a 22 Ch rigid cystoscope with a camera head and light source, a 0.035-inch straight hydrophilic guidewire, a 4.8 Fr straight ureteral catheter, a 60 mL Luer-lock syringe prefilled with diluted contrast medium, and additional sterile gloves. The upper table is reserved for the mini-PCNL phase and includes a sterile C-arm drape, a 0.035-inch guidewire, a 12 Fr semi-rigid nephroscope with 6° optics, a 16 Fr 13 cm Clear Petra suction sheath, a stone-collection container, a Holmium:YAG laser with a 550 µm fiber, and a nephrostomy set comprising fascia dilators up to 14 Fr, an 18 G Chiba needle, and an 8 Fr nephrostomy catheter. The procedure begins with placing a 4.8 Fr × 74 cm ureteral catheter into the PUJ over a guidewire using a 22 Fr Storz rigid cystoscope. An 18 Fr Foley catheter is then inserted for bladder drainage. The ureteral catheter is used to perform retrograde pyelography and guide tract dilation for percutaneous puncture. The percutaneous phase is performed under ultrasound and fluoroscopic guidance. After successful puncture, a guidewire is advanced into the ureter, and progressive dilation is performed up to 14 Ch. The inner mandrel of the 10/12 Ch sheath is then used to introduce a second safety guidewire. The Clear Petra sheath is inserted and connected to a suction system via a dedicated stone-collection reservoir. All dilations, including sheath placement, are performed under continuous fluoroscopic monitoring. Once the stone is visualized, lithotripsy is performed using a 550 µm Holmium laser fiber. As fragmentation proceeds, stone fragments are aspirated through the Clear Petra system. Upon completion of lithotripsy, the sheath is removed, and an 8 Fr nephrostomy tube is positioned in the renal pelvis. The ureteral catheter is connected to the Foley catheter for continuous drainage. The Foley catheter is removed on POD1, and the nephrostomy is typically removed on POD2.

### 2.4. Cost Analysis

A cost analysis was conducted to compare the economic impact of RIRS and mini-PCNL from the hospital’s perspective. The analysis included both direct and indirect costs associated with each procedure, following standard micro-costing methodology [11]. Direct costs encompassed all single-use disposable materials, OR occupancy time, and surgical and anesthesia personnel resources. Because the same institutional OR cost per minute was applied to both techniques, differences in OR-related costs reflect differences in operative time rather than differences in cost assumptions. The disposable instruments used for each procedure were identified through the hospital’s procurement database and priced using institutional purchase costs. The OR time costs were calculated by multiplying the average duration of each procedure by the institutional per-minute rate, which accounts for staffing, anesthesia, and overhead expenses. Indirect costs included LoS, perioperative imaging, and ancillary pre- and postoperative care. Hospitalization costs were estimated using the average daily inpatient expenditure for urological procedures in the Italian National Health Service [9]. Minor perioperative costs, such as laboratory tests, medications, and drainage management (<EUR 200 per case), were included in the total cost per procedure. However, these costs were excluded from the adjusted cost per treated and stone-free patient, as they were considered fixed and similar across procedures. They would proportionally influence all repeated interventions without changing the relative cost differences between techniques. Imaging costs for preoperative and postoperative non-contrast CT scans were covered and calculated using institutional tariffs, with an estimated cost of approximately EUR 175 per scan, resulting in a total imaging cost of EUR 350 per patient for pre- and postoperative evaluation. The total cost per patient was determined by summing direct and indirect costs. For patients requiring multiple interventions to become SF, the total cost was adjusted proportionally to the average number of procedures per patient. All costs were calculated in euros (€) and based on 2023 institutional prices. Costs related to sterilization and reprocessing of reusable instruments were not itemized separately; they are incorporated into institutional operating room overhead and applied uniformly across procedures. A detailed breakdown of unit costs and cost assumptions is provided in Supplementary Table S1.

### 2.5. Statistical Analysis

Statistical analyses were conducted using SPSS version 25 (IBM Corp., Armonk, NY, USA). Continuous variables were compared between groups with either the independent Student’s *t*-test or the Mann–Whitney U test, depending on the data distribution. Categorical variables were analyzed using the chi-square test or Fisher’s exact test as appropriate. Results are presented as mean ± standard deviation (SD) for continuous variables and as absolute numbers with percentages for categorical data. A two-sided *p*-value < 0.05 was deemed statistically significant. To adjust for baseline differences in stone size, a multivariable logistic regression was performed, with final stone-free status as the outcome and treatment modality and stone size as covariates.

## 3. Results

### 3.1. Baseline Characteristics and Postoperative Outcomes

A total of 119 patients were included in the study: 62 who underwent RIRS (Group A) and 57 who underwent mini-PCNL (Group B). The mean age was 62.5 years in Group A and 63.2 years in Group B (*p* = 0.132). The proportion of female patients was comparable between the groups (45% vs. 47% in groups A and B, respectively, *p* = 0.111). The mean BMI was 22.6 kg/m^2^ (SD 3.56) for Group A and 23.1 kg/m^2^ (SD 4.29) for Group B (*p* = 0.098).

The prevalence of major comorbidities was comparable between groups. In Group A, hypertension was present in 37.1% of patients, compared with 38.6% in Group B (*p* = 0.76). Diabetes mellitus was reported in 16.1% of patients undergoing RIRS and in 15.8% of those treated with mini-PCNL (*p* = 0.84).

Preoperative Hb levels averaged 13.1 g/dL (SD 0.93) in group A and 12.6 g/dL (SD 0.99) in group B (*p* = 0.144). Preoperative serum creatinine was 1.14 mg/dL (SD 0.34) in Group A and 1.16 mg/dL (SD 0.22) in Group B (*p* = 0.112). The mean preoperative WBC count was 7.2 × 10^3^/µL (SD 0.48) in Group A and 7.4 × 10^3^/µL (SD 0.83) in Group B.

In Group A, 59.7% of patients were classified as ASA II, 33.9% as ASA III, and 6.5% as ASA IV. Similarly, in Group B, ASA II, III, and IV patients accounted for 61.4%, 33.3%, and 5.3% of cases, respectively. The mean stone Dmax was 21.9 mm (3.94) in group A and 24.4 mm (SD 4.61) in group B (*p* = 0.003). The mean stone density was 1031 HU (SD 217.3) and 1058 HU (SD 197.7) in Groups A and B, respectively (*p* = 0.322).

The presence of multiple adjacent stones (clustered KS located within the same calyceal system or renal segment) was observed in 8.1% of patients in Group A and 4.0% in Group B. In Group A, KS were located in the left kidney in 46.8% of cases and in the right kidney in 53.3%, whereas in Group B, left-sided KS accounted for 56.1% and right-sided stones for 43.9% of cases (all *p* > 0.05).

Similarly, the anatomical distribution of stones within the renal collecting system was balanced between groups. In Group A, KS were located in the renal pelvis or PUJ in 9.7%, in the upper calyx in 30.6%, in the middle calyx in 25.8%, and in the lower calyx in 33.9%. In Group B, the corresponding proportions were 10.5%, 28.1%, 29.8%, and 31.6%, respectively, with no statistically significant differences across locations (all *p* > 0.05) (Table 1).

### 3.2. Postoperative Outcomes

Mean operative time was significantly shorter in the RIRS group compared with the mini-PCNL group (87 ± 12.97 vs. 113 ± 15.25 min; *p* < 0.001). Conversely, the length of hospital stay was significantly longer after mini-PCNL (2.24 ± 0.46 vs. 1.22 ± 0.26 days; *p* = 0.008). At 24 h postoperatively, HB levels were significantly lower in Group B (10.7 ± 1.53 g/dL) compared to Group A (12.2 ± 0.95 g/dL; *p* = 0.032). No significant differences were observed in postoperative serum creatinine (1.14 ± 0.28 vs. 1.20 ± 0.37 mg/dL; *p* = 0.232) or WBC count (13.1 ± 3.42 vs. 13.4 ± 2.67 × 10^3^/µL; *p* = 0.187). The mean number of procedures per patient was significantly higher in Group A (1.68 ± 0.52) than in Group B (1.35 ± 0.27; *p* < 0.001). Postoperative complications were comparable between groups and were predominantly low-grade according to the Clavien–Dindo classification. In Group A, 16 patients experienced Clavien–Dindo grade I complications, mainly transient postoperative fever or mild pain managed conservatively. One patient developed a grade II complication (febrile UTI) requiring intravenous antibiotic therapy. One grade IIIa complication occurred due to postoperative bleeding, which was successfully managed with selective angioembolization. In the mini-PCNL group, 17 grade I complications were recorded, primarily consisting of self-limiting fever or minor postoperative discomfort. Two patients experienced grade II and required blood transfusions. One patient developed a grade IIIb complication related to significant postoperative hemorrhage, which required surgical exploration for hematoma evacuation and hemostasis and was successfully managed. No patients in either group required admission to the intensive care unit. There were no procedure-related mortalities. Stone-free outcomes differed significantly between the two techniques. After the first procedure, the CIRF-SFR was 43.55% in Group A and 70.17% in Group B (*p* < 0.0001). After completion of treatment, the final CIRF-SFR was 88.71% for RIRS and 94.74% for mini-PCNL (*p* = 0.043). The absolute SFR remained significantly higher in the mini-PCNL group (82.45% vs. 74.2%; *p* = 0.035) (Table 2).

### 3.3. Multivariable Analysis

Given the significant baseline difference in stone size between groups, a multivariable logistic regression analysis was performed to evaluate factors independently associated with final stone-free status. The model included treatment modality (RIRS vs. mini-PCNL), stone size (Dmax), stone density, and stone location (lower vs. non-lower pole). In multivariable analysis, treatment modality was not independently associated with final stone-free status (OR = 2.35, 95% CI = 0.47–11.69; *p* = 0.296). Stone size was also not independently associated with stone-free outcome (OR 1.01 per mm increase, 95% CI 0.84–1.21; *p* = 0.933). In contrast, higher stone density was independently associated with a lower likelihood of achieving stone-free status (OR 0.53 per 100 HU increase, 95% CI 0.35–0.79; *p* = 0.002). Stone location (lower vs. non-lower pole) was not independently associated with stone-free status (adjusted OR 0.77, 95% CI 0.17–3.42; *p* = 0.730). Table 3 summarizes these findings.

### 3.4. Cost Analysis

#### 3.4.1. Direct Costs

Disposable materials accounted for the largest share of costs in both groups. Based on the consumables listed in Table 4, the total cost of disposable materials for a standard procedure was EUR 1097 for RIRS and EUR 806.42 for mini-PCNL (*p* = 0.004).

Mini-PCNL had a longer OT (113 min) compared with RIRS (87 min). Using an average cost of EUR 3.9 per operative minute, the estimated OR-related expense was EUR 340 for RIRS and EUR 440 for mini-PCNL (*p* = 0.012). Both procedures required similar preoperative and postoperative imaging, consisting of two non-contrast CT scans per patient, with a total imaging cost of approximately EUR 350.

#### 3.4.2. Indirect Costs

**Figure 3:**
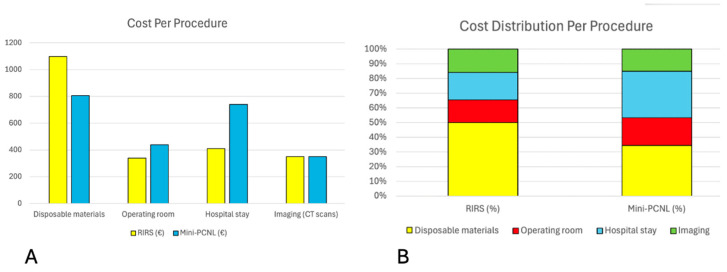
(**A**) Breakdown of direct and indirect cost components per procedure for RIRS and mini-PCNL operating room, hospital stay, and imaging. (**B**) Relative distribution of cost components expressed as a percentage of the total procedural cost.

Hospitalization accounted for an additional portion of total expenses. The mean LoS was 1.22 days for RIRS and 2.24 days for mini-PCNL. Based on an estimated inpatient cost of 334 EUR/day, there was an average hospitalization cost of approximately EUR 410 for RIRS and EUR 740 for mini-PCNL (*p* = 0.0001). Other pre- and postoperative costs, including laboratory tests, consultations, and drainage management, were considered minor and estimated at less than EUR 200 per patient. These expenses were not included in the adjusted cost calculations. When these expenditures were added, the total cost per procedure was EUR 2197 for RIRS and EUR 2336 for mini-PCNL (*p* = 0.138). Figure 3 displays the direct and indirect costs per procedure.

#### 3.4.3. Re-Intervention Costs

The average number of procedures per patient was 1.68 for RIRS and 1.35 for mini-PCNL. The total cost per treated patient, after adjusting for the number of procedures, was approximately EUR 3689 for RIRS and EUR 3150 for mini-PCNL (*p* = 0.009). When total costs were adjusted for the final SFR, the estimated cost per SF patient was EUR 4159 for RIRS and EUR 3331 for mini-PCNL (*p* = 0.007). When accounting for absolute SFR, the cost per stone-free patient increased to EUR 4971 for RIRS and EUR 3825 for mini-PCNL. The cost analysis summary is presented in Table 5.

## 4. Discussion

The current EAU guidelines describe PCNL as the procedure of choice for KS ≥ 20 mm, and it is still recommended for stones with a Dmax ≥ 10 mm [8,12]. However, recent technological advances in endoscopy and the development of miniaturized PCNL systems have made both procedures safer and more effective [13,14,15,16]. Consequently, the choice between RIRS and mini-PCNL remains controversial for KS between 10 and 20 mm, where outcomes are comparable but differ in invasiveness and costs [17,18]. Hence, we aimed to compare these two procedures with respect to perioperative outcomes and other clinical manifestations, while also considering their direct and indirect costs. Consistent with the previous literature, our results showed that mini-PCNL achieved a higher SFR than RIRS. The SFR is a critical parameter in endourology, as residual fragments increase the risk of recurrence and the need for retreatment [17,18]. In the present study, SFR was primarily defined as ≤4 mm, a threshold widely used in the endourological literature. However, this approach may overestimate stone-free rates relative to stricter definitions requiring the complete absence of residual fragments, and spontaneous passage of small residual fragments, particularly after RIRS, may not be fully captured at the 30-day follow-up; this should be considered when interpreting and comparing outcomes across studies. Because stone size differed significantly between treatment groups, an adjusted analysis was performed to address the potential impact of baseline stone characteristics on stone-free outcomes. Multivariable logistic regression showed treatment modality was not independently associated with stone-free status after adjustment, cautioning against overinterpreting the higher unadjusted stone-free rate with mini-PCNL. Stone size was not an independent predictor, implying baseline differences alone did not explain success. Stone density, however, was a predictor, with higher density linked to lower clearance. Stone location, modeled as lower versus non-lower pole, was not independently associated. These findings emphasize that crude stone-free rate comparisons may be influenced by stone characteristics and highlight the importance of adjusted analyses in retrospective studies. While mini-PCNL had higher unadjusted rates and cost-efficiency due to fewer retreatments, the adjusted analysis indicates that treatment success depends on multiple factors, not just the procedure type. De et al. [19] reported that standard PCNL achieved higher SFRs than RIRS; however, it was associated with greater blood loss and longer hospitalization. In contrast, Cabrera et al. showed that mini-PCNL provided an optimal balance between efficacy and invasiveness in stones 10–20 mm [16]. Jiang et al. [15], however, did not observe significant differences in SFR between micro-PCNL and RIRS, whereas stone size, location, and anatomy were associated with treatment success. In this study, as expected, OT and LoS were longer for mini-PCNL, reflecting the greater invasiveness of the percutaneous approach. However, complication rates were comparable between the two techniques, with most events classified as Clavien–Dindo grade I–II. The rates of postoperative fever and urosepsis in both groups were comparable to those reported in the literature [20,21,22,23,24,25,26]. In this study, one patient in group A developed postoperative bleeding that was successfully treated with selective angioembolization. Another patient in the mini-PCNL group experienced a significant postoperative hemorrhage that required exploratory laparoscopy for the evacuation of the hematoma and hemostasis. Both events have been described as possible bleeding complications following endourological procedures [20,27,28]. In addition to clinical outcomes, understanding the economic impact of endourological procedures has become increasingly important. The growing adoption of minimally invasive techniques, single-use devices, and expensive disposables necessitates precise cost assessment using micro-costing methods, which account for each resource individually rather than relying on aggregate reimbursement rates [29,30]. In this study, disposable instruments represented the principal cost driver, particularly in RIRS, where single-use ureteroscopes increased per-procedure expenditure. Previous cost analyses suggest that consumables account for approximately 40–60% of the total cost of flexible ureteroscopy, depending on volume and reprocessing practices [31,32,33,34]. In high-volume centers, the per-case cost of reusable flexible ureteroscopes ranges from USD 1212 to USD 1743 [33]. In contrast, the main costs in PCNL are driven by OT and LoS [35,36]. Leow et al. reported that longer procedures and extended admissions were associated with higher total expenditure [36]. In our analysis, the single-procedure cost was EUR 2197 for RIRS and EUR 2336 for mini-PCNL. When adjusted for the average number of procedures per patient, the total cost per treated patient increased to EUR 3689 for RIRS and EUR 3154 for mini-PCNL. Further adjustment for the final SFR yielded a cost per SF patient of EUR 4159 for RIRS and EUR 3331 for mini-PCNL. While mini-PCNL entails more complex surgery and a longer LoS, its higher per-session success rate offsets these costs, resulting in better overall cost-effectiveness. Each additional RIRS procedure increases expenses for anesthesia, imaging, and staff, which accounts for the higher total cost per successfully treated patient. Although the single-procedure cost of mini-PCNL was higher than that of RIRS, the overall cost-efficiency advantage of mini-PCNL was driven by a higher single-session stone-free rate and a lower retreatment rate. Therefore, the economic benefit observed in the present study reflects treatment success per session rather than lower procedural costs. These results align with studies by Cabrera et al. and Kumar et al. [16,17], who found that PCNL has higher single-session success rates and lower retreatment rates than RIRS, both of which significantly affect total procedure costs and resource use. However, economic trends and reimbursements vary across healthcare systems. In the United States, PCNL is typically more expensive than RIRS [37,38]. In European public healthcare systems, fixed personnel costs and high disposable-use rates make RIRS comparatively costly, particularly when single-use ureteroscopes are used. Under the Italian National Health Service, reimbursement is based on DRG, which assigns higher tariffs to PCNL than to RIRS [10]; however, these tariffs do not accurately reflect real costs. The micro-costing approach that we utilized seems to provide a more precise estimate of actual hospital expenditure, highlighting that mini-PCNL achieves superior cost-efficiency despite a longer procedure and LoS. This cost analysis is based on institutional prices and assumptions that might differ across healthcare systems. The relative cost-effectiveness of RIRS and mini-PCNL could change in settings where reusable flexible ureteroscopes are used, where OR minute costs vary significantly, or where length-of-stay costs are lower. However, because the primary cost benefit of mini-PCNL in our study was a lower retreatment rate rather than lower per-procedure costs, the overall conclusions are likely to remain valid across plausible cost scenarios. Recent studies have emphasized that cost-efficiency should be considered alongside clinical efficacy in selecting the optimal treatment modality. The PUrE trial demonstrated that mini-PCNL achieved higher single-session clearance and marginally superior cost-effectiveness for lower-pole stones within the British National Health Service [39]. Future studies should focus on validating these results in larger, prospective, multicenter cohorts to reduce selection bias and to account for regional differences in costs and clinical practices. Using standardized micro-costing methods across different institutions would improve comparability and establish cost benchmarks for endourological procedures. Including patient-reported outcomes, quality-of-life data, and indirect societal costs would provide a more comprehensive view of true cost-effectiveness. Additional research should investigate how innovations such as digital and single-use ureteroscopes, miniaturized PCNL systems, and AI-assisted surgical planning impact both clinical outcomes and economic sustainability.

## 5. Limitations

This study has several limitations worth noting. Its retrospective nature may have introduced selection bias, particularly because mini-PCNL was introduced only after April 2022, whereas RIRS procedures occurred throughout the study period. As a result, the choice of procedure was influenced more by the availability of the PCNL program and institutional logistics than by random assignment. Mini-PCNL was introduced during the study period, and therefore, a time-related selection bias cannot be completely excluded, as procedure allocation was influenced by equipment and personnel availability. However, all procedures were performed by surgeons with extensive prior experience in PCNL (>150 cases for each surgeon), making a clinically relevant operator learning-curve effect unlikely. Although the baseline characteristics appeared similar, unmeasured factors such as stone complexity or anatomical differences could have affected the outcomes. The small sample size and single tertiary center setting may also limit the generalizability of these findings. The cost analysis was conducted from the hospital’s perspective and did not account for indirect societal costs, such as productivity losses or postoperative recovery time. Furthermore, patient-centered outcomes, including postoperative pain, time to return to normal activities, and quality-of-life measures, were not systematically collected and could not be analyzed in the present study. This represents an important limitation, particularly in the context of cost-effectiveness evaluations, as indirect costs and patient-reported outcomes may substantially influence the overall value of different treatment strategies. Additionally, long-term costs such as stone recurrence or outpatient management were not examined. Finally, variations in device prices and inpatient expenses across different healthcare systems could influence cost comparisons.

## 6. Conclusions

In summary, both mini-PCNL and RIRS are safe and effective options for treating KS ≥ 15 mm. Mini-PCNL tends to have higher single-session stone-free rates but involves longer OT and longer LoS. However, it is more cost-effective per patient SF when retreatment rates are considered. These results emphasize the need to integrate clinical and economic assessments when selecting the optimal treatment for individual patients. Further prospective, multicenter randomized trials that include patient-reported outcomes and long-term cost analysis are necessary to validate these findings and optimize procedural guidelines.

## Figures and Tables

**Table 1:** Baseline patient demographics, comorbidities, and stone characteristics stratified by study group. Continuous variables are reported as mean ± standard deviation (SD), while categorical variables are expressed as percentages.

Variable	Group A (n° 62)	Group B (n° 57)	*p*
***Patient Characteristics***			
Age, years (SD)	62.5 (5.14)	63.2 (6.12)	0.132
Sex F, %	45	47	0.111
BMI, kg/m^2^ (SD)	22.6 (3.56)	23.1 (4.29)	0.098
Hypertension, %	37.1	38.6	0.76
Diabetes Mellitus, %	16.12	15.8	0.84
Metabolic syndrome, %	22.58	26.31	0.53
Hb pre-op, g/dL (SD)	13.1 (0.93)	12.6 (0.99)	0.144
Pre-op serum creatinine, mg/dL (SD)	1.14 (0.34)	1.16 (0.22)	0.112
Pre-op WBC, 10^3^/µL (SD)	7.2 (0.48)	7.4 (0.83)	0.244
ASA II, %	59.7	61.4	0.92
ASA III, %	33.85	33.34	0.98
ASA IV, %	6.45	5.26	0.83
***Stone Characteristics***			
Average Stone Dmax (SD)	21.9 (3.94)	24.1 (4.61)	0.003
Multiple adjacent stones, %	8.06	4	0.43
Mean Stone density “HU” (SD)	1031 (217.3)	1058 (197.7)	0.322
**Stone location, %**			
-Left Kidney	46.77	56.14	0.21
-Right Kidney	53.33	43.86	0.23
-Renal Pelvis/UPJ	9.67	10.52	0.86
-Upper Calyx	30.6	28.07	0.72
-Middle Calyx	25.8	29.84	0.58
-Inferior Calyx	33.93	31.57	0.79

**Table 2:** Postoperative outcomes. Data are reported as mean ± standard deviation (SD) for continuous variables and as absolute numbers or percentages for categorical variables.

Variable	Group A (n° 62)	Group B (n° 57)	*p*
Mean OT, minutes (SD)	87 (12.97)	113 (15.25)	<0.001
Mean LoS (SD)	1.22 (0.26)	2.24 (0.46)	0.008
Hb 24H post-op, g/dL (SD)	12.2 (0.95)	10.7 (1.53)	0.032
Serum creatinine 24H post-op, mg/dL (SD)	1.2 (0.37)	1.14 (0.28)	0.232
24H post-op WBC, 10^3^/µL (SD)	13.4 (3.42)	13.1 (2.67)	0.187
Clavien–Dindo I	16	17	
Clavien–Dindo II	1	2	
Clavien–Dindo IIIa	1	0	
Clavien–Dindo IIIb	0	1	
Average N° of procedures per patient (SD)	1.68 (0.52)	1.35 (0.27)	<0.001
SFR 1°, %	43.55	70.17	<0.0001
SFR 2°, %	88.71	94.74	0.043
Absolute SFR, %	74.2	82.45	0.035

**Table 3:** Multivariable logistic regression model evaluating the association between treatment modality and final stone-free status.

Variable	β	Adjusted OR	95% CI (Lower–Upper)	*p*
Constant	9.26	-	-	-
Procedure (mini-PCNL vs. RIRS)	0.86	2.35	0.47–11.69	0.296
Stone size (Dmax, per 1 mm)	0.01	1.01	0.84–1.21	0.933
Stone density (per 100 HU)	−0.64	0.53	0.35–0.79	0.002
Lower pole location (yes vs. no)	−0.26	0.77	0.17–3.42	0.730

**Table 4:** List of disposable materials and their cost per unit in Euros.

Consumable Materials Used for Standard Procedure	RIRS (Unit N°)	PCNL (Unit N°)	Cost per Unit (€)
Sensor Guidewire	1	2	120
Rocamed Dual-Lumen Sheath	1	1	117
Pusen Flexible Ureteroscope	1	0	800
JJ Coloplast Stent	1	0	60
Clear Petra Nephrostomy	0	1	360
Clear Petra Collection Bottle	0	1	10
Coloplast Ureteral Catheter	0	1	5.90
Rocamed Nephrostomy	0	1	73.52
**Total, €**	1097	806.42	

**Table 5:** Breakdown of direct and adjusted costs and mini-PCNL.

Cost Component (€)	RIRS	Mini-PCNL	*p*
Disposable materials	1097	806	0.004
OR cost	340	440	0.012
LoS	410	740	0.001
Imaging	350	350	N/A
Total cost per procedure	2197	2336	0.138
Average procedures per patient	1.68	1.35	0.021
Total cost per treated patient, €	3689	3154	0.009
SFR, %	88.7	94.7	0.043
Cost per stone-free patient, €	4159	3331	0.007
Absolute SFR, %	74.2	82.45	0.035
Cost per absolute stone-free patient, €	4971	3825	0.002

## Data Availability

Data availability is restricted due to privacy or ethical reasons imposed by the Institution but might be available upon reasonable request.

## Supplementary Materials

The following supporting information can be downloaded at https://www.mdpi.com/article/10.3390/jcm15010177/s1, Table S1. Unit costs and cost assumptions used in the micro-costing analysis.

## Abbreviations

The following abbreviations are used in this manuscript:

AUA	American Urological Association
BMI	Body mass index
CIRF	clinically insignificant residual fragments
CT	Computed tomography
Dmax	Maximum stone diameter
DRG	Diagnosis-related group
EAU	European Association of Urology
ESWL	Extracorporeal shock wave lithotripsy
Hb	Hemoglobin
HDI	Human Development Index
HU	Hounsfield unit
ICOT	Istituto Chirurgico Ortopedico Traumatologico
LoS	Length of stay
mini-PCNL	Mini-percutaneous nephrolithotomy
NHS	National Health Service
OR	Operating room
OT	Operative time
PCNL	Percutaneous nephrolithotomy
POD	Postoperative say
PUJ	Pyelo-ureteral junction
QoL	Quality of life
RIRS	Retrograde intrarenal surgery
SD	Standard deviation
SF	Stone-free
SFR	Stone-free rate
URS	Ureteroscopy
WBC	White blood cell count
