# Supplementary material for: Comparison Between RIRS and Mini-PCNL in the Treatment of Kidney Stones Exceeding 15 mm: Outcome Evaluation and Cost Analysis
Source: J Clin Med. 2025 Dec 26;15(1):177. doi: 10.3390/jcm15010177 (PMC12786861; doi:10.3390/jcm15010177)
Supplement: Supplementary file 1 [file jcm-15-00177-s001.zip › jcm-4039189-supplementary.pdf]

Supplementary Table S1. Unit costs and cost assumptions used in the micro-costing analysis

Cost item	Unit cost (€)	Unit	Source / Assumption	Included in total cost per procedure	Included in adjusted cost (treated/SF)
Operating room time	3.9	€/min	Institutional OR accounting	✓	✓
Hospital stay	334	€/day	Italian NHS average tariff	✓	✓
CT scan (non-contrast)	175	€/scan	Institutional tariff	✓	✓
Pre + post CT	350	€/patient	2 × CT	✓	✓
Disposable flexible ureteroscope	800	€/unit	Hospital procurement database	✓	✓
Dual-lumen sheath	117	€/unit	Hospital procurement database	✓	✓
Guidewire	120	€/unit	Hospital procurement database	✓	✓
Double-J stent	60	€/unit	Hospital procurement database	✓	✓
Clear Petra sheath	360	€/unit	Hospital procurement database	✓	✓
Nephrostomy tube	73.52	€/unit	Hospital procurement database	✓	✓
Minor perioperative costs*	<200	€/case	Estimated average	✓	✗
Reusable instrument processing	—	—	Included in OR overhead	✓	✓

* Minor perioperative costs include laboratory tests, perioperative medications, and drainage management. These costs were considered fixed and comparable across procedures and were therefore excluded from adjusted cost calculations per treated and stone-free patient.
